# Uncovering the Absolute Structure of the Malarial Pigment Crystal, Hemozoin, Using Cryo-electron Tomography and Three-Dimensional Electron Diffraction

**DOI:** 10.1021/acscentsci.4c01267

**Published:** 2024-08-15

**Authors:** Renaldo Sutanto, Shyamal Mosalaganti

**Affiliations:** † Life Science Institute, University of Michigan, Ann Arbor, Michigan 48109, United States; ‡ Department of Cell and Developmental Biology, University of Michigan, Ann Arbor, Michigan 48109, United States; § Department of Biophysics, College of Literature, Science and the Arts, University of Michigan, Ann Arbor, Michigan 48109, United States; ∥ Department of Biological Chemistry, University of Michigan, Ann Arbor, Michigan 48109, United States

Malaria is a potentially fatal disease caused by *Plasmodium* parasites transmitted to humans by infected mosquitoes. The infection process begins in the liver and subsequently targets red blood cells, which are rich in hemoglobin. The parasite internalizes the cytoplasm of these blood cells, transporting it to its acidified digestive vacuole, where various enzymes break down hemoglobin to supply essential biomolecules for the parasite’s growth and survival. Since the heme component of hemoglobin is toxic to the parasite, it converts heme into nontoxic, insoluble hemozoin crystals as a means of detoxification. Existing antimalarial drugs target this exact growth process, inhibiting the formation of hemozoin, which results in the fatal accumulation of free heme within cells.^1^

Our understanding of how antimalarial drugs interact with early stage or developing hemozoin crystals to slow their growth has advanced. Nevertheless, much of what we know about the crystal structure of hemozoin has been extrapolated from studies on its synthetic counterpart, β-hematin. Prior research has postulated that the effective binding of drugs to the crystal’s surface is contingent upon specific chemical interactions.^2^ In this issue of *ACS Central Science*, Klar et al. provide the structure and polarity of biogenic hemozoin crystals, known both historically and diagnostically as the pigments associated with malaria.^3^ The findings presented in this paper make a vital contribution by carefully characterizing the absolute crystal structure, polar morphology, and growth patterns of biogenic hemozoin. This research is not only of foundational scientific significance but is also vitally relevant to medical knowledge, particularly in the ongoing battle against malaria.

Hemozoin crystals, isolated from ruptured *Plasmodium falciparum* cells, exhibit a distinct polar morphology and are more regular in shape and size than their synthetic counterparts. These biogenic hemozoin crystals feature deeply corrugated (101) and (1̅01̅) surfaces. Tomographic reconstruction of the digestive vacuole containing these crystals reveals that one end displays a smooth, chisel-like shape, while the other is markedly irregular.

One of the most remarkable discoveries in this study is the asymmetry between the two monomers in the hemozoin crystal lattice. Electron diffraction analysis, combined with kinematical modeling, identifies a 1:1 mixture of dimers—one centrosymmetric (R/S′) and one chiral (R/R′)—with atomic disorder observed in the −*c* and +*b* orientations. This mixed-isomer scenario is further validated by density functional theory (DFT) calculations, which show that the R/S′ and chiral dimer mixture is more energetically favorable compared to two chiral enantiomers or two centrosymmetric dimers. In essence, the authors unambiguously resolve a long-standing debate on the composition of isomers (1:1 of R/S′ and R/R′, respectively) in the lattice and provide validation that this can co-crystallize with minimal energetic penalties.

The authors also uncover atomic disorder on certain crystal facets, characterized by their asymmetric nature. Utilizing transmission electron microscopy (TEM), Klar et al. imaged the crystals to reveal the detailed lattice structures, including the arrangement of methyl-vinyl pairs on various surfaces. Furthermore, they observed that the carboxyl and carboxylate groups are exposed to the aqueous medium on the (001̅) and (001) surfaces, making them hydrophilic. Consequently, the crystal growth of the (001̅) and (001) faces are inhibited by competitive water binding, which influences the crystal’s growth dynamics. Ultimately, this phenomenon accounts for the distinctive morphological differences between the opposite ends of biogenic hemozoin crystals.

Since biogenic hemozoin accumulates in the digestive vacuole, it might incorporate organic macromolecules like neutral lipids, cholesterol, or proteins from its complex growth environment, potentially causing distortions or defects in the crystal lattice. However, this hypothesis seems inconsistent with the high-resolution structure observed from crystallographic analysis. Mosaicity measurements from diffraction data revealed a median misorientation of just 0.08°, which is relatively small compared to other organic and inorganic crystals. In addition, 4D scanning transmission electron microscopy (4D STEM) measurements showed consistent mass density and composition, with a slight tilt (1°–2°) and a minor bending strain in rod-like crystals. Therefore, these tests found no evidence of dislocations, voids, or other defects indicative of foreign macromolecule incorporation, supporting the conclusion that hemozoin is pure crystal.

Utilizing an array of structural techniques, Klar et al. have meticulously detailed the architecture of native hemozoin from *Plasmodium falciparum*. This breakthrough paves the way for intriguing research opportunities, particularly in understanding the dynamic interactions between antimalarial drugs and the initiation of biogenic hemozoin. Key questions, such as the precise dimerization mechanism that enables hemozoin to select the correct orientation for crystal lattice growth without inhibition, remain to be answered. Biochemical studies suggest that heme detoxification proteins (HDPs) selectively promote this dimerization process.^4,5^ However, high-resolution structural data are still lacking to definitively show how these proteins bind to heme monomers, enhancing the formation of one chiral dimer over another. HDPs potentially serve as chiral templates, promoting the assembly of dimer forms optimized for rapid crystallization—a process crucial to the parasite’s survival. Moreover, understanding how common antimalarial drugs interact with biogenic hemozoin and disrupt crystal growth is of paramount importance. Future directions could entail using this sophisticated structural blueprint of hemozoin to design targeted antimalarial therapies, potentially marking a novel approach in the fight against malaria, and to craft treatments that precisely interfere with the parasite’s detoxification pathway.
